# Influences of Differentiated Residence and Workplace Location on the Identification of Spatiotemporal Patterns of Dengue Epidemics: A Case Study in Guangzhou, China

**DOI:** 10.3390/ijerph192013393

**Published:** 2022-10-17

**Authors:** Yuqi Zhang, Hongyan Ren, Runhe Shi

**Affiliations:** 1 State Key Laboratory of Geographic Information Science, Ministry of Education, East China Normal University, Shanghai 200241, China; 2 Institute of Geographic Sciences and Natural Resources Research, Chinese Academy of Sciences, Beijing 100101, China; 3 School of Geographic Sciences, East China Normal University, Shanghai 200241, China; 4 Key Laboratory of Spatial-Temporal Big Data Analysis and Application of Natural Resources in Megacities, Ministry of Natural Resources, Shanghai 200241, China; 5 Joint Laboratory for Environmental Remote Sensing and Data Assimilation, ECNU&CEODE Ministry of Education, East China Normal University, Shanghai 200241, China

**Keywords:** dengue fever, differentiating workplace, residence locations, GIS, spatiotemporal patterns

## Abstract

The location of the infections is the basic data for precise prevention and control of dengue fever (DF). However, most studies default to residence address as the place of infection, ignoring the possibility that cases are infected at other places (e.g., workplace address). This study aimed to explore the spatiotemporal patterns of DF in Guangzhou from 2016 to 2018, differentiating workplace and residence. In terms of temporal and spatial dimensions, a case weight assignment method that differentiates workplace and residence location was proposed, taking into account the onset of cases around their workplace and residence. Logistic modeling was used to classify the epidemic phases. Spatial autocorrelation analysis was used to reveal the high and early incidence areas of DF in Guangzhou from 2016 to 2018. At high temporal resolution, the DF in Guangzhou has apparent phase characteristics and is consistent with logistic growth. The local epidemic is clustered in terms of the number of cases and the time of onset and outbreak. High and early epidemic areas are mainly distributed in the central urban areas of Baiyun, Yuexiu, Liwan and Haizhu districts. The high epidemic areas due to commuting cases can be further identified after considering the workplaces of cases. Improving the temporal resolution and differentiating the workplace and residence address of cases could help to improve the identification of early and high epidemic areas in analyzing the spatiotemporal patterns of dengue fever in Guangzhou, which could more reasonably reflect the spatiotemporal patterns of DF in the study area.

## 1. Introduction

Dengue fever (DF) is an acute mosquito-borne disease caused by the dengue virus and occurs mainly in urbanized and semi-urbanized areas in tropical, subtropical, and even warm temperate zones around the world [1,2]. More than 100 countries worldwide are affected by DF, with approximately 390 million people infected with dengue virus each year [2,3]. Currently, DF has been classified as the most serious insect-borne disease in the world by the World Health Organization [4].

The DF outbreak in China is a typical imported epidemic [5,6,7,8]. With the acceleration of globalization and increasingly frequent international exchanges in China, the risk of local DF epidemics in China induced by the imported disease has increased significantly and has formed a typical epidemic area with a frequent and high incidence of DF in southern China [9,10,11]. Among them, Guangzhou is one of the most serious areas of DF in China [5,6,12]. In 2014, Guangzhou experienced one of the largest DF epidemic in more than 20 years, with a record high of 45,224 reported cases [13]. In recent years, with the rapid urbanization of population, land, and urban space elements, and the continuous improvement of the public transportation system, the population aggregation and mobility in urban areas have continued to increase. As a result, the DF epidemic in Guangzhou has increased in both prevalence and transmission rate [14,15,16,17].

Characterization and understanding of the spatiotemporal distribution and transmission patterns will provide theoretical support for DF prevention and control [18,19,20,21]. Previous studies have raised our understanding of its epidemiological characteristics and spatiotemporal patterns, by which high epidemic areas were identified for making timely interventions on this disease [22,23]. Fan et al. [24] explored the spatial distribution patterns of DF in Guangdong Province based on incidence rates at the district and county scales, and pointed out the high epidemic areas; Khormi et al. [25] mapped DF risk based on the Getis–Ord Gi* method and frequency index to explore the changes in DF incidence hotspots from a long-term perspective. All of these studies provided a basis for precise DF prevention and control from the perspective of identifying high incidence areas of the epidemic. However, DF outbreaks often spread initially from an epidemic site and caused different scales of transmission. To detect and interrupt the spread of early epidemics is critical for reducing the emergence of high epidemic areas. Therefore, more attention should be paid to these special areas, where the outbreak occurs early and spreads outward. Monitoring early epidemic areas not only can control the outward spread of the epidemic promptly but is also important for tracing the source of the epidemic. However, existing studies on early epidemic monitoring focus more on temporal warnings [26], whilst few have been conducted on early epidemic areas.

In the human–mosquito–human transmission patterns of DF, due to the limited flight distance of the mosquito vector, daily movement of people is one of the important factors of the widespread spread of DF within cities, in addition to local environmental factors that trigger large-scale outbreaks [27,28,29]. Although previous studies have paid attention to the influence of human activities on dengue transmission [30,31,32,33,34,35], to characterize the spatiotemporal association patterns between specific population dynamics characteristics and DF patterns often requires informative human movement data to support. However, we recognized that the availability of such data is not high in some regions [36], limiting the development of related studies. We thus attempt to find data or information that characterizes human mobility and meanwhile avoids the limitations of data availability, that is, the information based on residence addresses and workplace addresses. As a national legal category B infectious disease, cases in the direct network reporting system contain information on residential address and work address, which provides refined case data for the study of the spatiotemporal distribution patterns of DF. In addition, it has been demonstrated that a person may become infected at home or other places where he or she stays for a long time (e.g., workplace), suggesting home and workplace as possible sites of infection [37]. Therefore, workplace address of the cases cannot be ignored in spatial analysis.

Therefore, this study was conducted to investigate the spatiotemporal distribution patterns of DF in Guangzhou City from 2016 to 2018 based on the case information differentiating residence and workplace location and model simulation of the temporal characteristics of epidemic development on a fine scale, and to reveal the high and early epidemic areas, with a view of providing reference for the understanding of the development characteristics of DF epidemic in typical areas and its precise prevention and control.

## 2. Materials and Methods

### 2.1. Study Area

**Figure 1:**
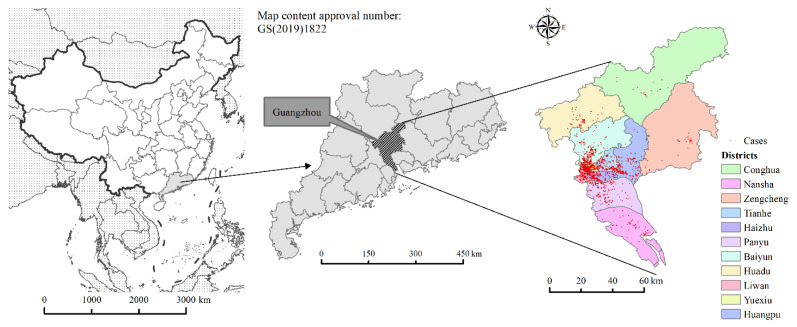
Location map of the study area. Red dots represent reported cases of Dengue Fever outbreaks from 2016 to 2018.

Guangzhou, with its high population density, foreign population, and mobility, is a typical epidemic area of DF in China. This paper takes Guangzhou as the study area. Guangzhou is located in the northern part of the Pearl River Delta of Guangdong Province (112°57′ E–114°03′ E, 22°26′ N–23°56′ N). It has 11 districts and 170 neighborhoods and towns, with a total area of 7434.40 km^2^ and a resident population of 18,676,600 in 2020. In this study, the neighborhoods and towns are taken as the smallest study unit. The location map of the study area is shown in Figure 1.

### 2.2. Epidemiological Data

The data of DF cases in Guangzhou from 2016 to 2018 were obtained from the Guangzhou Center for Disease Control and Prevention. The data was desensitized so that each case information contained only gender, age, time of onset, residence address, workplace address, and case type (imported or local cases). Imported cases refer specifically to the cases imported from abroad, i.e., cases that have traveled to countries or regions outside of China where DF is endemic within 14 days before the onset time. Local cases, however, refer to the cases that have not left the county (current residence address) within 14 days prior to the onset time. A total of 2104 cases were collected from 2016 to 2018, of which 1915 were local cases, 189 were imported cases, and 429 cases (20.39%) contained workplace addresses. The cases were spatially located based on the case residence and workplace address and the geocoding service provided by Baidu Open Platform.

**Figure 2:**
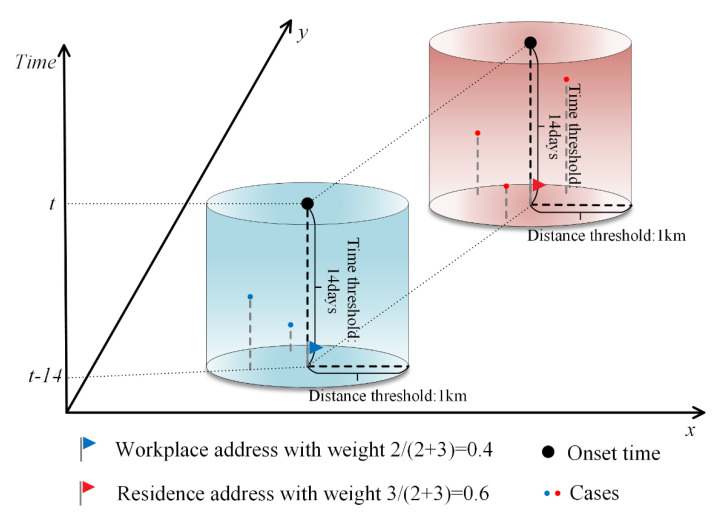
Schematic diagram of the method of assigning weights to workplace and residence addresses.

According to the distribution characteristics of activity time in a day for urban residents in Guangzhou City, for those who have a job, the time they spend in the residence and workplace has the highest percentage of the whole day. Most activities in these two locations are also relatively stationary, and people are more likely to be bitten by mosquitoes. Therefore, in order to more objectively reflect the location of infection of cases, this study assumed that cases could be infected at and near both workplace and residence locations, but due to the uncertainty, it was necessary to assign 0–1 weights to workplace and residence addresses according to the associated cases around the workplace and residence addresses, respectively. For cases with both workplace and residence addresses, a circular buffer zone with a radius of 1 km was established at the center of their workplace and residence addresses, respectively (determined according to the activity radius of the 15-min living circle in Guangzhou [38]). The number of cases in the buffer zone was counted 14 days before the onset time of the case (determined according to the longest incubation period of DF [39]), and the weights of workplace and residence addresses were assigned according to the ratio of the number of cases meeting both spatial and temporal buffer conditions. For cases containing only residence addresses, the residence address weight is set to 1 and the workplace address weight is set to 0. The number of cases was obtained by summing the weights of the points of workplace and residence locations within the study area. The schematic diagram of the method of assigning weights to workplace and residence addresses is shown in Figure 2.

### 2.3. (Population) Logistic Growth Model

(Population) logistic growth model describes the development process of something that shows S-shaped growth due to resource and environmental constraints, and was originally used to study the development patterns of biological populations [40]. Similarly, the growth process of DF cases also satisfies this law due to the influence of the seasonal pattern of mosquito vectors and the duration of transmission.

The logistic differential equation model is:

(1) $$\frac{dy}{dt}=ry\left(1-\frac{y}{K}\right)$$

The logistic integral form is:

(2) $$Y\left(t\right)=\frac{a}{1+b\cdot e^{-ct}}$$

where *Y*(*t*) is the cumulative number of DF cases, *t* is the time variable (weeks), *K*, *a*, *r* are constants, *K* is the limit value of the cumulative number of cases, *a* is the integration constant, and *r* is the growth rate.

By solving Equation (2) for the second and third order derivatives respectively, we can obtain the three inflection points of the model, whose corresponding time points are:

(3) $$t_{m}=\frac{lnb}{c}$$

(4) $$t_{2}=\frac{lnb-1.317}{c}$$

(5) $$t_{3}=\frac{lnb+1.317}{c}$$

*t_m_* is the point of maximum case growth rate, and *t*_2_, *t*_3_ are the points of maximum rate of change of case growth rate. The number of weeks for the first and last case onset of the corresponding case type in that year was noted as *t*_1_ and *t*_4_, respectively. Based on the above points of temporal characteristics, the DF epidemic can be divided into the initial onset phase (*t*_1_*–t*_2_), the middle outbreak phase (*t*_2_*–t*_3_), and the late receding phase (*t*_3_*–t*_4_) [40,41,42]. Note that *t*_1_ is the time of onset, *t*_2_ is the time of outbreak, *t*_3_ is the time of receding, and the duration of *t*_2_ to *t*_3_ is lasting time.

The coefficient of determination *R*^2^ and F-test were used to test the goodness of fit and significance

(6) $$R^{2}=1-\frac{\sum_{i=1}^{n}{\left(E_{i}-O_{i}\right)}^{2}}{\sum_{i=1}^{n}{\left(\bar{O_{i}}-O_{i}\right)}^{2}}$$

(7) $$F=\frac{\frac{\sum_{i=1}^{n}{\left(O_{i}-\bar{O_{i}}\right)}^{2}}{n-1}}{\frac{\sum_{i=1}^{n}{\left(E_{i}-\bar{E_{i}}\right)}^{2}}{n-1}}$$

where *O_i_* is the actual cumulative number of cases in week *i*, *E_i_* is the number of fitted cases in week *i*, and *n* is the number of samples. The larger R^2^, the better the curve fit is. If *F* < *F*0.05, it indicates that the curve fitting effect is significant at 0.05 confidence level, and *F*0.05 is obtained by checking the F-value distribution table at the degree of freedom *f* = *n* − 1.

In this study, logistic growth curves were fitted for dengue cases in the whole city of Guangzhou and 170 neighborhoods and towns separately to extract temporal characteristic points and classify the epidemic phases. Logistic curve fitting was implemented by MATLAB R2019a platform.

### 2.4. Spatial Autocorrelation Analysis

Spatial autocorrelation is an effective tool to study regional aggregation of infectious diseases and to perform epidemic analysis [43,44], which is based on spatial location attributes to analyze whether a phenomenon (e.g., morbidity) between adjacent regions is spatially correlated, i.e., whether there is a spatial aggreation of infectious diseases [45]. It can be divided into global and local autocorrelation. In this study, spatial autocorrelation analysis will be conducted based on the number of cases and the temporal characteristics of the epidemic phases in the whole of Guangzhou and each neighborhood and town.

In this study, the global Moran’s I was used to detect the spatial distribution patterns of the number of cases and the temporal characteristic points of each phase of the epidemic in Guangzhou, and to reveal whether there was an aggregation of the DF epidemic in Guangzhou in number and time and the strength of autocorrelation. The global Moran’s I was calculated as follows.

(8) $$I=\frac{n}{\sum_{i=1}^{n}\sum_{j=1}^{n}w_{ij}}\times \frac{\sum_{i=1}^{n}\sum_{j=1}^{n}w_{ij}\left(x_{i}-\bar{x}\right)\left(x_{j}-\bar{x}\right)}{\sum_{i=1}^{n}{\left(x_{i}-\bar{x}\right)}^{2}}$$

where *n* is the number of townships with DF outbreaks, *X_i_* and *X_j_* denote the number of cases (sum of the weights of workplace and residence locations) or temporal characteristic (*t*_1_*, t*_2_*, t*_3_, lasting time) of neighborhood and town *i* and *j*, respectively. *W_ij_*(*d*) is the spatial adjacency weight matrix at distance *d*. For neighborhood and town *i* and *j*, this *W_ij_*(*d*) is 1 if *i* and *j* are adjacent and 0 if they are not. Moran’s I is between −1 and 1. A high positive Moran’s I value with larger z-score and/or appropriate *p*-value represents a tendency towards clustering, which means that adjacent units have similar incidence rates, whereas a low negative value indicates a tendency towards dispersal, which means that units with high incidence rates lie next to units with low incidence rates.

The global Moran’s I is a comprehensive measure of spatial autocorrelation for the whole study area, which can only indicate the average degree of spatial differences between each unit and the surrounding area; but there may be a coexistence of partial spatial positive correlation and partial spatial negative correlation in the overall study area, and thus the local spatial autocorrelation statistic is needed to reveal possible spatial variability. In this study, the local Moran’s I, which calculates the number of DF cases and the temporal characteristics of the epidemic in each neighborhood and town, was used to detect the type of spatial aggregation of DF in Guangzhou in terms of quantity and time, so as to explore the high and early epidemic areas with practical significance. The local Moran’s I was calculated as follows.

(9) $$I_{i}=\frac{x_{i}-x}{\frac{\sum_{j=1,j\neq i}^{n}{\left(x_{j}-x\right)}^{2}}{n-1}-x^{2}}\times \overset{n}{\underset{j=1,j\neq i}{\sum }}w_{ij}\left(x_{j}-x\right)$$

The meaning of the symbols is the same as Formula (7). The clustering and outlier classification can be divided into high value (HH) clusters with a significance level of 0.05, low value (LL) clusters, high value outliers surrounded mainly by low values (HL), and low value outliers surrounded mainly by high values (LH).

For the number of cases, the HH aggregation area indicates the area with a higher number of incidences. As for the temporal characteristics, since the number of weeks was used as an attribute value to measure the start time of each phase, HH aggregation area indicated areas with later dengue outbreaks, while LL aggregation area indicated areas with earlier outbreaks and deserved to be focused on. Therefore, in this study, the HH aggregation area with the number of cases was defined as the high epidemic areas, and the LL aggregation area with the temporal characteristics (the time of onset and the time of outbreak) was defined as the early epidemic areas. The above spatial autocorrelation analysis was implemented through ArcGIS 10.6 platform.

## 3. Results

### 3.1. Epidemiological Characteristics

The population statistics of DF cases in Guangzhou from 2016–2018 are shown in Table 1. The number of male cases in both local and imported cases was slightly larger than that of female case (*χ*2 = 7.15, *p* < 0.05; *χ*2 = 14.86, *p* < 0.05). The population of cases was mainly concentrated in the group of young people aged 19–45. The gender difference in DF may be due to the different occupations of the different genders with exposure differences. In addition to the different daily movement patterns of the older and younger people, the age difference in DF is also related to the fact that the older people may have been previously infected with DF and thus may have resistance to it [46]. The occupations of local cases were mainly domestic and household and unemployed (22.35%), commercial service (17.81%) and blue-collar worker (14.93%), while the occupations of imported cases were mainly commercial service (22.22%), domestic and household and unemployed (20.11%) and unknown (14.29%). Except for household and unemployed, retired, other and unknown, all other occupational groups were considered as commuting cases, and the percentage of commuting cases among local cases was 52.54%, i.e., more than half of the cases could have been infected at their workplace. Therefore, the workplace should be added to the spatial analysis as a possible site of infection.

### 3.2. Phases of the Epidemic

**Figure 3:**
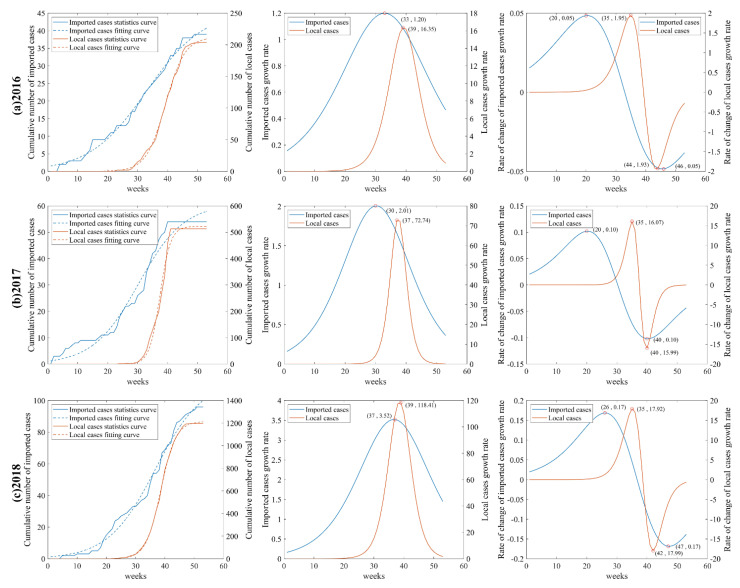
Logistic growth curves and first and second order derivatives of imported and local cases of DF, 2016–2018 (**a**−**c**).

The logistic growth curve fitting expressions and the evaluation of the goodness of fit are shown in Table 2. The logistic growth curves and the first-order and second-order derivatives are shown in Figure 3a–c. The logistic growth fitting curves all passed the F-test, and the fit was good.

The preliminary statistics of cases and the results of the growth curve fitting revealed that the DF epidemic is consistent with logistic growth and has obvious phase characteristics at high temporal resolution. The time phases of the DF epidemic from 2016 to 2018 are shown in Table 3. The beginning of the middle outbreak phase of the imported cases from 2016 to 2018 were 20 weeks, 20 weeks, and 26 weeks, respectively, and the beginning of the middle outbreak phase of local cases were all 35 weeks, all lagging behind the imported cases. These results suggest that although the phase characteristics of DF varies from year to year, imported cases are always an important factor for the spread of local epidemic.

### 3.3. Global Spatial Autocorrelation

**Figure 4:**
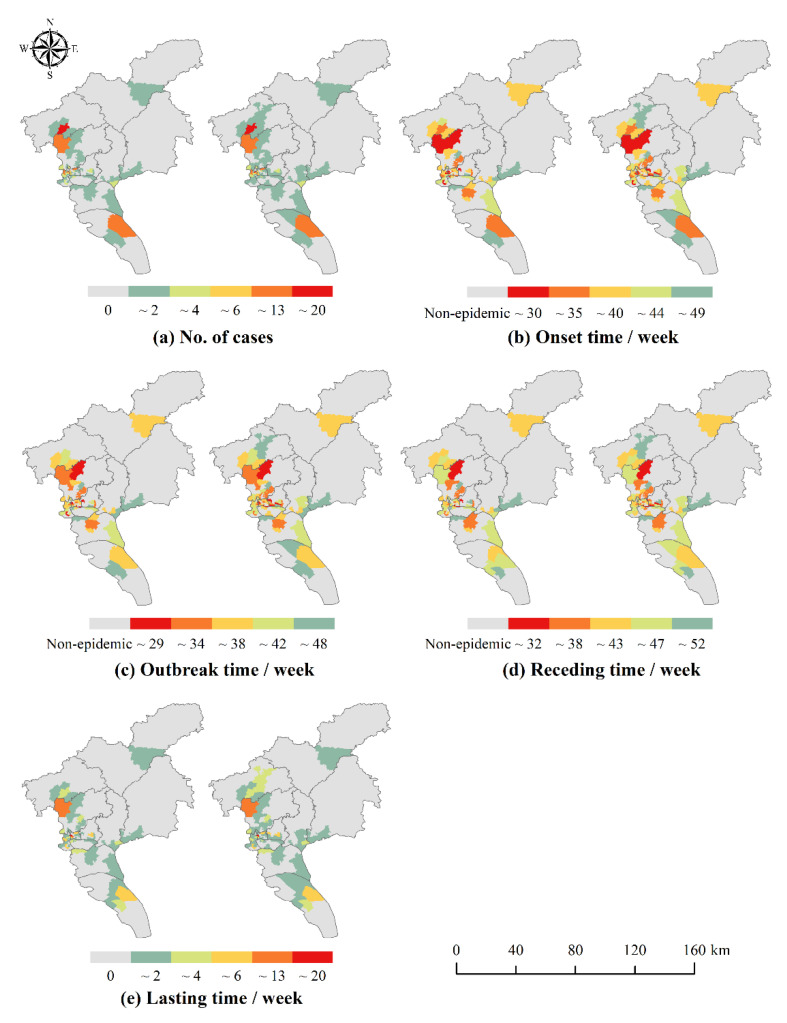
Distribution of the number of DF cases (**a**) and time characteristics of DF cases (**b**–**e**) in 2016. **left**: With non-workplace: without differentiating workplace and residence location; **right**: With workplace: differentiating workplace and residence location.

**Figure 5:**
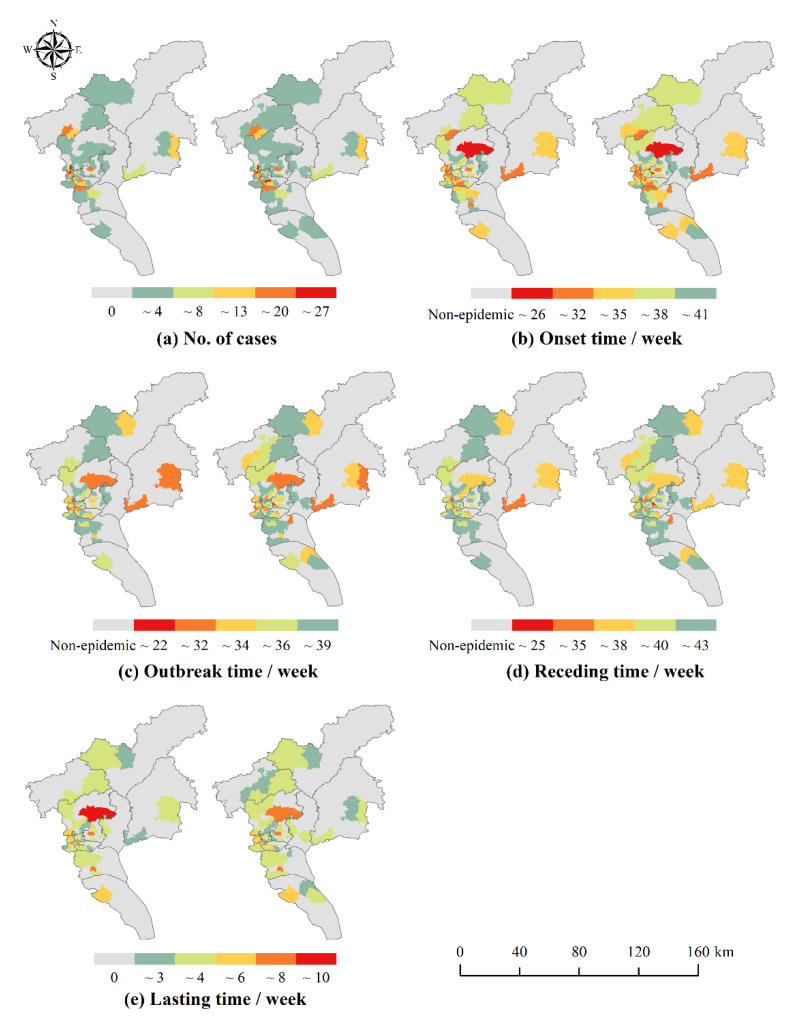
Distribution of the number of DF cases (**a**) and time characteristics of DF cases (**b**–**e**) in 2017. **left**: With non-workplace: without differentiating workplace and residence location; **right**: With workplace: differentiating workplace and residence location.

**Figure 6:**
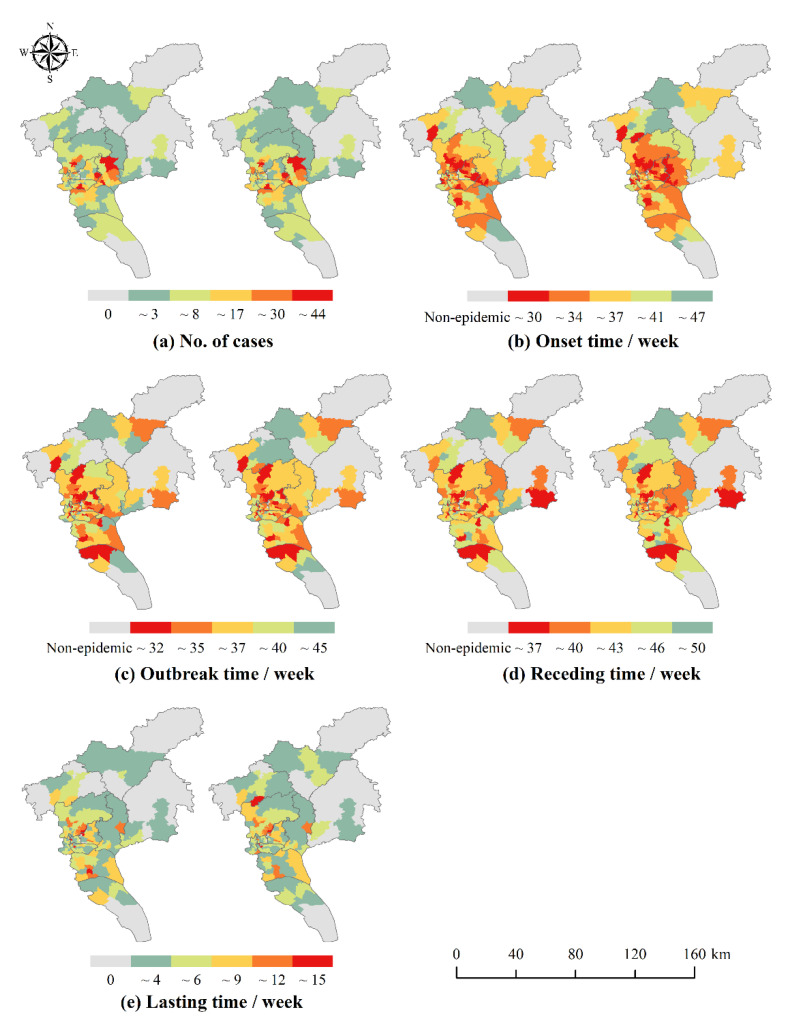
Distribution of the number of DF cases (**a**) and time characteristics of DF cases (**b**–**e**) in 2018. **left**: With non-workplace: without differentiating workplace and residence location; **right**: With workplace: differentiating workplace and residence location.

The distribution of the number of local cases and the temporal characteristics of each phase in Guangzhou from 2016 to 2018 are shown in Figure 4, Figure 5 and Figure 6. There were slight spatial differences in the number of local cases and temporal characteristics of each phase in Guangzhou City with or without differentiating workplace and residence addresses (with workplace and with non-workplace). When taking into account the workplace address, the range of neighborhoods and towns with local cases expanded. Most of the neighborhoods and towns changed from 0 cases to a small number of cases, and a few other neighborhoods and towns showed a significant increase in the number of cases, such as some neighborhoods and towns in Yuexiu District in 2016, Haizhu District in 2017, and Baiyun and Huangpu Districts in 2018. The onset, outbreak and receding of the epidemic in some neighborhoods and towns were advanced, for example, some neighborhoods and towns in Tianhe District in 2016 and 2017, and Huadu District, Baiyun District and Huangpu District in 2018.

The results of spatial autocorrelation analysis are shown in Table 4. Whether or not to differentiate workplace and residence address can affect the spatial autocorrelation of the number of cases and the onset time of DF. In terms of the number of cases, there was a significant spatial autocorrelation of DF in Guangzhou, and in 2016 and 2018, the Moran’s I that differentiated workplace and residence increased slightly compared to the Moran’s I that did not. The aggregation of DF in Guangzhou was more obvious after considering the possibility of infection at workplace. This is because, after differentiating workplace and residence, there are more possible places of infection once considering the workplace address of the cases, thus making the distribution of cases more clustered. In terms of temporal characteristics, the clustering distribution of DF onset and outbreak time from 2016 to 2018 was more obvious, and the receding time and lasting time were more randomly distributed. Thus, the number of cases and the onset and outbreak time of DF were significantly clustered throughout the study area.

### 3.4. Identification of Early and High Epidemic Areas

**Figure 7:**
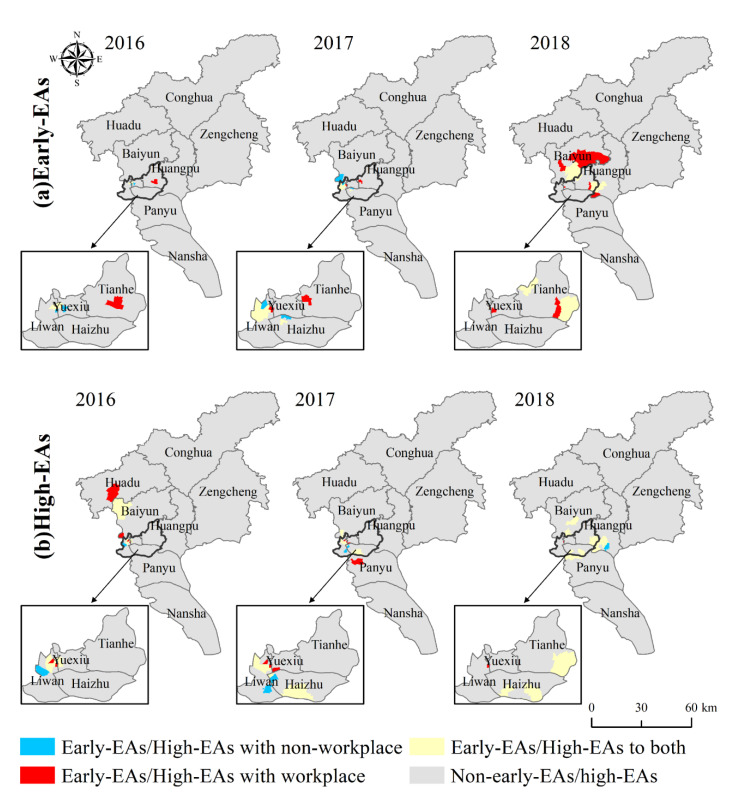
Distribution of DF early-EAs (**a**) and high-EAs (**b**) in Guangzhou. Early-EAs: early epidemic areas; High-EAs: high epidemic areas; With non-workplace: without differentiating workplace and residence location; With workplace: differentiating workplace and residence location.

The early (early-EAs) and high (high-EAs) epidemic areas whether or not differentiating workplace and residence of DF local epidemic in Guangzhou from 2016 to 2018 are shown in Figure 7a,b. The statistical results are shown in Table 5. Both early-EAs and high-EAs obtained based on whether or not to differentiate workplace and residence addresses differed in spatial terms. With workplace, new early epidemic neighborhoods and towns were found in Tianhe District and new high epidemic neighborhoods and towns were found in Liwan District in both 2016–2018. Overall, the early and high epidemic neighborhoods and towns of DF in Guangzhou in 2016–2018 were mainly distributed in the five administrative districts of Yuexiu, Liwan, Haizhu, Tianhe, and Baiyun, and the central city was a high-risk area for the occurrence and outbreak of DF.

Comparing the statistical results of high-EAs whether or not differentiating workplace and residence, there are more identical high-EAs and a small number of different high-EAs, with a total of 8 identical high-EAs in 2016, 9 identical high-EAs in 2017, and 15 identical high-EAs in 2018. The statistical results of different high-EAs are shown in Table 6.

With workplace, the weight of residence address is weakened because the workplace address of cases is taken into account, the number of cases in high-EAs with non-workplace is reduced, and some high-EAs become non-high-EAs. Within the high-EAs with workplace, the number of cases increased in Xiuquan Neighborhood in 2016, Renmin Neighborhood, Rainbow Neighborhood, and Dashi Neighborhood in 2017, and Longjin Neighborhood in 2018 after differentiating workplace and residence. Except for Dashi Neighborhood (2017) and Longjin Neighborhood (2018), the number of commuting cases in all other neighborhoods and towns accounted for a greater proportion than the cases that included workplace addresses (20.39%). Therefore, the increase of commuting cases was the main reason why these neighborhoods and towns became high-EAs. In other high-EAs with workplace, the number of cases in each neighborhood and town decreased or remained unchanged but the number of cases in their neighboring neighborhood and town increased due to the inclusion of the weight of workplace address, based on the corresponding local Moran’s I and *p* value, thus judging the neighborhood or town as a high-EA as well. Thus, it is necessary and feasible to differentiate workplace and residence for the identification of high-EAs.

Identifying early-EAs is important to prevent aggravation and spread of the epidemic. According to the statistical results of early-EAs, the onset time of epidemic in early-EAs was 30–32 weeks, and the outbreak time was 33–34 weeks. Comparing the statistical results of early-EAs whether differentiating workplace and residence, there were three identical early-EAs in 2016, 7 identical early-EAs in 2017, and 13 identical early-EAs in 2018. For early-EAs identified based on local autocorrelation analysis, if the epidemic was not effectively controlled in time and developed into high-EAs later, the neighborhood or town was considered as a potential high-EA. Meanwhile, the outbreak time of the early-EA was compared with the outbreak time (week 35) of the epidemic in Guangzhou, and if outbreak time ≤ 35, it was proved to be correct to determine the neighborhood or town as an early-EA. The statistical results of the early-EAs with outbreak time ≤35 weeks and the potential high-EAs whether differentiating workplace and residence are shown in Table 7. For the identification of potential high-EAs, the percentage of the number of neighborhoods and towns with workplace was higher than the percentage of those with non-workplace, and they were all above 50%, except for 2018. Therefore, it is necessary and feasible to differentiate workplace and residence for the identification of potential high-EAs. For the identification of neighborhoods and towns with outbreak time ≤35 weeks, the weight distribution method of differentiating workplace and residence is generally effective, and the accuracy rate decreases compared with that of not differentiating workplace and residence. The identification of early-EAs, differentiating workplace and residence yet needs to be further explored.

## 4. Discussion

In this study, we proposed a case weight assignment method differentiating the DF cases’ workplace and residence addresses, simulated the epidemic growth curves at the week intervals by means of a logistic model, and then identified the stages and corresponding areas with early and high epidemics in Guangzhou City, thus confirmed the necessity and feasibility of the method and provided a reference for conducting similar studies and prevention and control guidance.

This study found that the DF epidemic in Guangzhou has obvious phase characteristics and is consistent with logistic growth. There were small numbers of the cases in the initial onset phase of the epidemic, which grew slowly, and most were imported cases. The number of cases in the middle outbreak phase increased significantly and the development of the epidemic became slow. With the decreasing number of cases in the late receding phase, the epidemic leveled off. In addition, there was a lag in local cases compared to imported cases. The initial phase of epidemic was mainly dominated by imported cases, and local cases gradually increased as imported cases introduced dengue virus to the mainland. The middle and late phases of the outbreak were mainly dominated by local cases, while the high mobility of imported cases and untimely diagnoses may have become contributing factors to the spread of the local epidemic outbreak. The above conclusions are consistent with the findings of Chen et al. [47] and have corresponding theoretical bases in the research methodology. A precise division of the epidemic phases is of great importance to objectively describe the development pattern of the epidemic and to precisely guide the prevention and control of the epidemic. Since it takes time to implement health decisions and interventions, early warnings tend to be with lags if allowing the epidemic to develop to the middle outbreak phase. Therefore, it is recommended to conduct early warnings with timely decisions to implement relevant epidemic prevention measures 1–2 weeks before the middle outbreak phase.

The number of DF cases and the onset time and outbreak time of DF epidemic in Guangzhou from 2016 to 2018 were significantly clustered. Previous studies revealed the epidemic clusters by spatiotemporal scan analysis [48,49,50]. This study, however, conducted spatial autocorrelation analysis on the temporal characteristics of the epidemic in addition to considering the number of cases. It thus reveals the spatial and temporal distribution of the early and high epidemic areas of DF in Guangzhou, and provides a new perspective for DF risk monitoring. The high and early epidemic areas of DF in Guangzhou were mainly distributed in the central urban areas of Baiyun, Yuexiu, Liwan, and Haizhu districts, and the early epidemic areas spread from the central urban areas to the surrounding areas. This conclusion is similar to the results of the epidemiological characteristics analysis of DF in Guangzhou City by Liu et al. [51]. This may be related to the natural and socio-economic environment of the central urban area. Longjin Neighborhood, one of the high and early epidemic areas in 2016 and 2017, may be taken as an example. The neighborhood contains a large area of urban villages where the houses are mostly old buildings with shady and moist environments, and some areas have high building density, high population density and mobility, which are conducive to the breeding of *Aedes albopictus* and the spread of dengue virus. It is, therefore, recommended that the epidemic prevention departments should step up the efforts in such neighborhoods and towns in the central city.

The weight assignment method differentiating workplace and residences is a useful exploration of the spatial and temporal epidemiological patterns of DF, and it deserves further investigation, although its impact is not very ideal for identifying early epidemic areas. Urban residents have complex travel patterns. They can be bitten by mosquitoes at any time and any place and thus infected with dengue virus. To get the fine-grained human activity trajectory relies on movement big data. However, it is difficult either to obtain or to process because of the large amount of data. And since the human movement trajectories obtained through movement big data are based on the level of all urban residents, we cannot know which trajectories are left by infected case. Therefore, this study explored the spatiotemporal distribution patterns of DF at the level of individual cases, based on their workplace and residence information. For people who have a job, the time they spend at the workplace and residence in a day is about 8 hours and 12 hours respectively [52,53], which cannot take up the whole day but are the two highest percentage of time in a day. The probability of being bitten by mosquitoes is relatively high in these two locations where there are mostly stationary activities during the time. Therefore, this study integrates the possibility that cases may be infected within the workplace and residence locations and nearby environments that meet daily life, and assigns 0–1 weights to workplace and residence locations respectively according to the incidence. When comparing the high epidemic and early epidemic areas whether differentiating workplace and residence, there are a few neighborhoods and towns that are different from each other, in addition to most of the same neighborhoods and towns. For the high epidemic areas, the weight assignment method that differentiates workplace and residence can identify more high epidemic areas with a certain degree of confidence. Since the local Moran’s I reflects the aggregation situation among neighborhoods and towns, it is not only determined by the number of cases in individual neighborhoods and towns, but also influenced by the change of epidemic in neighboring neighborhoods and towns. As for the early epidemic areas, although the method identified different early epidemic areas, the overall accuracy rate was reduced. The possible reason is that under the premise that the number of cases in Guangzhou remains unchanged, the number of possible infection locations in space becomes more frequent leading to a smaller weight of each point, and some neighborhoods and towns are affected by this decrease in the total number of cases, which makes it difficult to form a complete time series and leads to errors when fitting logistic growth curves. In addition, for early cases, there may be few or even no cases in the 14 days before the onset time, and the weight assignment will be biased. Both of these situations can affect the judgment of early epidemic areas. Since the weight assignment method takes into account the possibility of infection at both residence and workplace, the resulting risk area is more comprehensive. Accordingly, it is recommended that the relevant epidemic prevention departments expand the scope of risk surveillance, focusing on the same high epidemic areas and not missing different high epidemic areas, while increasing the prevention efforts in the neighborhoods and towns around the high epidemic areas.

There are certain limitations in this study: (1) According to the available data statistics, the number of commuting cases accounts for 52.54%, while the number of cases with workplace addresses including case information only accounts for 20%. Although significant differences in the distribution of high epidemic areas can be found based on the available 20% data, which can meet the experimental requirements, overall it is still suggested that relevant departments should improve the collection and management system of case information to ensure the integrity and availability of the data. (2) The choice of both temporal and spatial buffers affects the results of the workplace and residence addresses weighting. In this study, only one spatial and temporal buffer was used. Especially for initial cases, in the absence of a priori information, the case weight assignment method, although logically reasonable, can be biased in practical applications. It can be further considered to explore the optimal workplace and residence addresses weighting scheme by adopting various combinations of spatial and temporal buffers according to different onset situations and a priori information. (3) There is no unified criterion for the definition of early epidemic areas. The early epidemic areas in this study were artificially defined based on the epidemic phases and local spatial autocorrelation analysis results, which inevitably had some subjectivity and thus affected the effect of early epidemic area identification. In the future, expert scoring method could be used to define the criteria for judging early epidemic areas. (4) The factors influencing the spatiotemporal distribution of DF are complex, and this study only explored the high and early epidemics of DF based on the epidemic data, and the causes and influencing mechanisms need to be further investigated. In the future, the association between DF patterns and risk factors could be explored using methods such as geographically weighted regression or the geographical detectors.

## 5. Conclusions

This study shows that high temporal resolution can help to determine the epidemic development pattern more accurately, and the weight assignment method that differentiates workplace and residential more objectively reflects the location of infection, both of which are significant in the study of spatiotemporal patterns of DF. This study provides a new method and perspective for the study of spatial and temporal patterns of DF, especially for the identification of high and early epidemic areas of DF. Therefore, it is suggested that relevant epidemic prevention departments should accurately grasp the key time points of the epidemic, make early warnings and decisions, and strengthen the collection of workplace address of cases.

## Figures and Tables

**Table 1:** The gender, age, and occupation distributions of DF cases in Guangzhou, 2016–2018.

Gender	Number of Local Cases	Percentage (%)	Number of Imported Cases	Percentage (%)
Male	1016	53.05	121	64.02
Female	899	46.95	68	35.98
**Age**				
0–18	132	6.89	7	3.70
19–45	1037	54.15	140	74.07
46–60	430	22.45	29	15.34
>60	316	16.50	13	6.88
**Occupation**				
Household and unemployed	428	22.35	38	20.11
Retired	267	13.94	10	5.29
Business Service	341	17.81	42	22.22
Cadre	109	5.69	18	9.52
Blue-collar worker	286	14.93	23	12.17
Farmer	75	3.92	9	4.76
Student	137	7.15	10	5.29
Child	25	1.31	0	0
Teacher	21	1.10	5	2.65
Medical Staff	12	0.63	3	1.59
Others	49	2.56	4	2.12
Unknown	165	8.62	27	14.29
Total	1915	100	189	100

**Table 2:** Logistic growth curve expressions and goodness of fit.

Year	Type of Case	Parameter Estimation			Goodness of Fit			Curve Fitting Expression
		a	b	c	R^2^	F-Value	F-Test	
2016	Imported	45.71	31.85	0.11	0.99	1.02	Pass	$y=\frac{45.71}{1+31.85e^{-0.11t}}$
	Local	212.18	185,284.76	0.31	1	1.00	Pass	$y=\frac{212.18}{1+185,284.76e^{-0.31t}}$
2017	Imported	60.78	54.54	0.13	0.98	0.95	Pass	$y=\frac{60.78}{1+54.54e^{-0.13t}}$
	Local	522.50	1,614,515,520.49	0.57	1	1.00	Pass	$y=\frac{522.50}{1+1,614,515,520.49e^{-0.57t}}$
2018	Imported	113.16	96.53	0.12	0.99	1.02	Pass	$y=\frac{113.16}{1+96.53e^{-0.12t}}$
	Local	1215.52	3,740,220.58	0.39	1	1.00	Pass	$y=\frac{1215.52}{1+3,740,220.58e^{-0.39t}}$

**Table 3:** Three phases of the DF epidemic in Guangzhou, 2016–2018.

Year	Type of Case	Initial Onset Phase/Week	Middle Outbreak Phase/Week	Late Receding Phase/Week
2016	Imported	4–19	20–46	47–49
	Local	23–34	35–44	45–51
2017	Imported	2–19	20–40	40
	Local	24–34	35–40	41
2018	Imported	5–25	26–47	48–51
	Local	22–34	35–42	43–50

**Table 4:** Results of global autocorrelation analysis of DF in Guangzhou.

Attributes	Year	With Non-Workplace				With Workplace			
		Moran’s I	Z Score	*p* Value	Results	Moran’s I	Z Score	*p* Value	Results
No. of cases	2016	0.20	4.73	0.00	Clustered (α = 0.01)	0.21	4.84	0.00	Clustered (α = 0.01)
	2017	0.24	5.31	0.00	Clustered (α = 0.01)	0.24	5.29	0.00	Clustered (α = 0.01)
	2018	0.35	7.78	0.00	Clustered (α = 0.01)	0.36	7.90	0.00	Clustered (α = 0.01)
Onset time	2016	0.09	0.91	0.36	Random	0.15	1.89	0.06	Clustered (α= 0.10)
	2017	0.18	2.34	0.02	Clustered (α = 0.05)	0.16	2.35	0.02	Clustered (α = 0.05)
	2018	0.20	4.01	0.00	Clustered (α = 0.01)	0.23	4.64	0.00	Clustered (α = 0.01)
Outbreak time	2016	0.18	1.73	0.08	Clustered (α = 0.10)	0.16	2.04	0.04	Clustered (α = 0.05)
	2017	0.19	2.51	0.01	Clustered (α = 0.05)	0.04	1.31	0.19	Random
	2018	0.08	1.60	0.11	Random	0.19	1.81	0.07	Clustered (α= 0.10)
Receding time	2016	0.07	0.74	0.46	Random	0.06	0.88	0.38	Random
	2017	0.17	2.27	0.02	Clustered (α = 0.05)	0.06	0.94	0.35	Random
	2018	0.03	0.68	0.49	Random	0.04	0.81	0.42	Random
Lasting time	2016	0.15	1.68	0.09	Clustered (α= 0.10)	−0.09	−1.02	0.31	Random
	2017	0.00	0.11	0.92	Random	0.01	0.28	0.78	Random
	2018	0.05	1.14	0.25	Random	0.01	0.24	0.81	Random

With non-workplace: without differentiating workplace and residence location; With workplace: differentiating workplace and residence location.

**Table 5:** Statistical results in high-EAs and early-EAs.

Type of Area	Year	With Non-Workplace			With Workplace		
		No. of Neighborhoods and Towns	No. of Cases	Proportion (%)	No. of Neighborhoods and Towns	No. of Cases	Proportion (%)
Early-EAs	2016	5	35	17.33	4	26.72	13.23
	2017	11	103	20.08	10	80.03	15.60
	2018	13	271	22.62	18	313.69	26.18
High-EAs	2016	9	42	20.79	12	66.10	32.72
	2017	11	112	21.83	13	110.40	21.52
	2018	16	306	25.54	16	299.05	24.96

Early-EAs: early epidemic areas; High-EAs: high epidemic areas; With non-workplace: without differentiating workplace and residence location; With workplace: differentiating workplace and residence location.

**Table 6:** Statistical results of different high-EAs whether differentiating workplace and residence.

Year	Type of Area	Neighborhood or Town	With Workplace			With Non-Workplace
			No. of Cases	No. of Commuting Cases	Percentage of Commuting Cases (%)	No. of Cases
2016	With non-workplace	Shiweitang Neighborhood (Liwan)	2.75	0.75	27.27	3
	With workplace	Longjin Neighborhood (Liwan)	8.71	0.5	5.74	9
		Caihong Neighborhood (Liwan)	13.02	0.75	5.76	13
		Xiuquan Neighborhood (Huadu)	1.5	0.5	33.33	1
		Jinsha Neighborhood (Baiyun)	3	0	0	3
2017	With non-workplace	Baihedong Neighborhood (Liwan)	3.3	0.08	2.42	4
		Longfeng Neighborhood (Haizhu)	10.83	0	0	11
	With workplace	Renmin Neighborhood (Yuexiu)	3.06	1.05	34.31	2
		Longjin Neighborhood (Liwan)	5.83	0	0	6
		Caihong Neighborhood (Liwan)	4.08	2.08	50.98	2
		Dashi Neighborhood (Panyu)	3.1	0.6	19.35	3
2018	With non-workplace	Hongshan Neighborhood (Huangpu)	6.5	0	0	8
	With workplace	Longjin Neighborhood (Liwan)	7.53	0.53	7.04	7

High-EAs: high epidemic areas; With non-workplace: without differentiating workplace and residence location; With workplace: differentiating workplace and residence location.

**Table 7:** Statistical results of early-EAs whether differentiating workplace and residence.

Year		2016	2017	2018
With non-workplace	No. of neighborhoods and towns in early-EAs	5	11	13
	No. of neighborhoods and towns in potential high-EAs	2	4	8
	Percentage (%)	40	36.37	72.73
	No. of neighborhoods and towns with outbreak time ≤35	4	11	11
	Percentage (%)	80	100	84.62
With workplace	No. of neighborhoods and towns in early-EAs	4	10	18
	No. of neighborhoods and towns in potential high-EAs	3	6	10
	Percentage (%)	75	60	55.56
	No. of neighborhoods and towns with outbreak time ≤35	2	9	12
	Percentage (%)	50	90	66.67

Early-EAs: early epidemic areas; High-EAs: high epidemic areas; With non-workplace: without differentiating workplace and residence location; With workplace: differentiating workplace and residence location.

## Data Availability

The data presented in this study are available on request from the corresponding author.
